# Intuitive eating as a counter-cultural process towards self-actualisation: An interpretative phenomenological analysis of experiences of learning to eat intuitively

**DOI:** 10.1177/20551029211000957

**Published:** 2021-03-10

**Authors:** Gabrielle A. Erhardt

**Affiliations:** Westminster University, UK

**Keywords:** biopedagogy, counter-cultural, interpretative phenomenological analysis, intuitive eating, self-actualisation

## Abstract

This research presents an in-depth idiographic study that illustrates how learning to eat intuitively involves socio-cultural challenges, strategies of resistance and self-actualising processes. Interviews were conducted with eight women who had been practising intuitive eating (IE) for at least 1.5 years. Data was analysed using IPA and four themes were drawn inductively from the data: IE as an ongoing process, perceived judgement of others, strategies of resistance and processes of self-actualisation. Further research is needed to explore experiences of learning to eat intuitively amongst different samples and with different cultures, and to further investigate the relationship between IE and the actualising tendency.

## Introduction

Intuitive eating (IE) has attracted increasing attention as an adaptive style of eating and an alternative to dietary restriction. Research demonstrating IE’s beneficial outcomes in relation to psychological and physical health has recently expanded, but there is very little qualitative research exploring how people become intuitive eaters. This research seeks to address this gap and contribute to the understandings of experiences of becoming an intuitive eater. Employing an inductive qualitative approach, this research provides a contextualised account of the lived experience of becoming an intuitive eater.

### Intuitive eating (IE)

**Figure 1.**
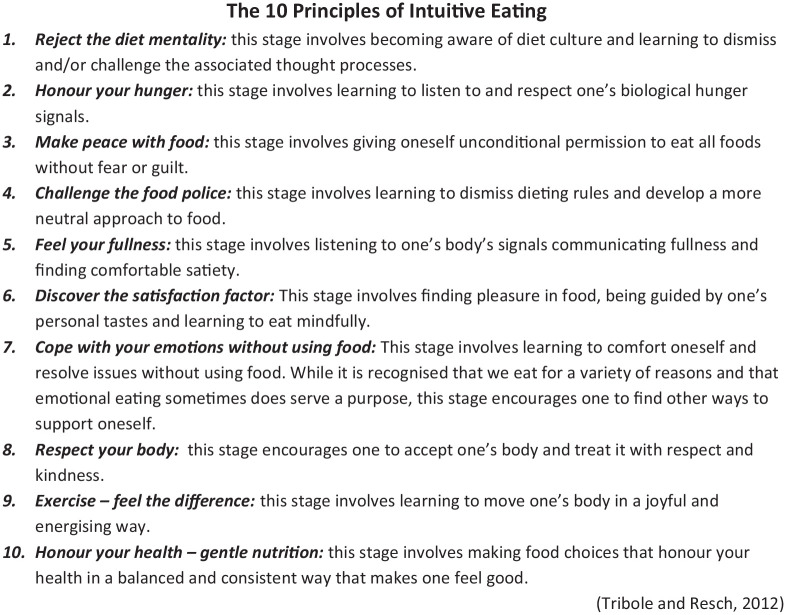
The 10 principles of intuitive eating.

IE is a non-diet weight inclusive approach that promotes eating based on internal cues, satisfaction and the health of mind and body (Tribole and Resch, 2012). It is an adaptive style of eating that focuses on the promotion of health independent of weight or dieting. Individuals who eat intuitively are not preoccupied with food and dieting and routinely respect their internal hunger and satiety cues (Augustus-Horvath and Tylka, 2011). As such, it is aligned with the Health at Every Size (HAES) movement which embraces a holistic definition of health as well as recognising the broader sociocultural environment (Watkins and Gerber, 2016). IE is generally based on 10 principles outlined by Tribole and Resch (2012) (see Figure 1.).

IE was developed in response to concerns regarding the efficacy and ethics of focusing on body weight and dieting. Evidence overwhelmingly asserts that weight loss is inaccessible and counterproductive for the vast majority of people. Research has repeatedly shown that dieting is associated with weight gain (National Institutes of Health, 1992) and according to Mann et al.’s (2007: 220) review, ‘one third to two thirds of dieters regain more weight than they lost on their diets’. In addition, dieting is a significant risk factor for eating disorders (Neumark-Sztainer et al., 2006), body dissatisfaction (Holm, 2007), osteoporosis (Bacon et al., 2004), psychological stress (Tomiyama et al., 2010), the adverse effects of weight cycling (Montani et al., 2006) and psychological problems including depression and decreased self-esteem (Ackard et al., 2002; Johnson and Wardle, 2005).

Research on IE is relatively limited since the majority of eating behaviour research has focused on pathology and maladaptive eating rather than adaptive eating practices (Avalos and Tylka, 2006). IE has been of particular interest to counselling psychologists whose adherence to phenomenological theory and a humanistic value system facilitate an approach focused on wellbeing rather than pathology (Milton, 2010). Thus far studies suggest that IE interventions result in improvements in psychological and physical wellbeing. Research has found that IE is inversely associated to eating disorder symptomology and positively associated with positive body image, self-esteem, positive emotional functioning, proactive coping, health promoting behaviours and overall life satisfaction (Bacon et al. 2005; Bruce and Ricciardelli, 2016; Sairanen et al., 2015). In addition, IE is associated with improved health risk indicators including metabolic fitness, reductions in the risk of heart disease and decreases in cholesterol, triglycerides and systolic blood pressure (Bacon et al., 2002, 2005; Hawks, 2004). Interestingly, but perhaps less meaningfully for a weight inclusive approach, IE is also associated with lower BMI (Denny et al., 2013).

### Becoming an intuitive eater

There is a distinct lack of qualitative research exploring people’s experiences of learning to eat more intuitively. While quantitative research shows that individuals can learn to eat more intuitively (Cole and Hoacek, 2007), it is unclear how this process happens or how this process is experienced. The handful of studies that do principally explore the process of IE, confuse IE with related but distinct practices such as mindful eating (e.g. Willig et al., 2014) or approach IE from a weight centric perspective (e.g. Wehling and Lusher, 2019). This is perhaps reflective of psychology more generally, a discipline that has been criticised for its adherence to a weight centric biopedagogy both within research and amongst applied psychologists (Bauman, 2020; Tylka et al. 2014).

An exception to this is Barraclough et al.’s (2019) thematic analysis exploring mid-aged women’s experiences of learning to eat intuitively in New Zealand. This research found that while participants were able to learn to eat more intuitively, they encountered ‘social and environmental barriers’ to IE, in particular a lack of understanding from friends and family (Barraclough et al., 2019: 1). Participants also explained how the process of learning to eat more intuitively resulted in a realignment of values and perceptions of the self (Barraclough et al., 2019). Both of these findings have been supported by quantitative research. Avalos and Tylka’s (2006) acceptance model of IE proposes that IE is one expression of the actualisation tendency. The actualising tendency is rooted in humanistic theory and asserts that people have a directional propensity to ‘express and activate all the capacities of the organism, or the self’ (Rogers, 1967: 351). IE’s prioritisation of inner experiences and bodily needs enables individuals to focus more on their needs and what they require to thrive, supporting self-actualisation. Research by Schoenefeld and Webb (2013) similarly suggests that IE is an example of embracing one’s values, thus alluding to the self-actualising tendency of IE. This actualising tendency, however, can be disrupted by external influences. While quantitative IE research tends to neglect the contextual factors involved in IE, Avalos and Tylka’s (2006) model highlights the importance of the perceived judgement of others and the corollary impact on self-acceptance and patterns of eating. This research found that women who perceive that others accept their body are more likely to accept their own body and engage in IE behaviours (Avalos and Tylka, 2006). The inverse was found regarding lack of acceptance by others.

Society’s role in shaping eating habits and perspectives on appearance has been widely documented (Culbert et al., 2015). In regard to IE, it has been proposed that one is born with the ability to eat intuitively, but the longevity of this eating style is dependent on environmental influences, in particular, the influence of ‘diet culture’ (Tribole and Resch, 2012). Diet culture is a dominant cultural discourse that is ‘predicated on the fear of fatness. . . and equates weight loss with health’ (Kinavey and Cool, 2019: 118). Diet culture hinges on neoliberal assertions of the autonomous human subject who is responsible and accountable for their own health (LaMarre et al., 2017). Research on eating disorder recovery has adopted Wright and Harwood’s (2012) theory of ‘biopedagogies’ (LaMarre and Rice, 2016). Drawing on Foucault’s (1978) notion of biopower, biopedagogies are pedagogies of *bios* that work to govern bodies and produce embodied subjectivities that instruct and form meaning. The imperative of weight loss is one such biopedagogy that shapes embodied subjectivities. This self-disciplining imperative has been interpretated by feminist scholars as a powerful tool of patriarchal discipline.

### This research

Given the long-term ineffectiveness and the risk of adverse effects of dieting, serious ethical concerns have been raised regarding recommending dieting for weight loss. IE provides a promising weight inclusive adaptive approach to eating and health. More research is required to investigate how individuals can be supported in the transition to this style of adaptive eating. This study seeks to add to the limited research on IE and further explore women’s experiences of learning to eat more intuitively, focusing in particular on the barriers and challenges faced by individuals during the process. Using Interpretative phenomenological analysis (IPA), this research adopts an inductive exploratory approach that attempts to illuminate individual subjective lived experience on its own terms.

### Exploratory research questions

- How do individuals experience the process of becoming an intuitive eater?
- What are the main barriers and/or challenges associated with intuitive eating?
- How are these barriers and/or challenges negotiated?

## Methodology

### Research design

This research employed a qualitative approach. Qualitative methodologies are well suited for conducting exploratory in-depth research that examines complex attitudes and contextual factors. Semi-structured interviews were conducted which provided a focus to the research while also supporting a flexible and explorative approach. IPA’s phenomenological approach lends itself well to the exploration of lived experience and the meanings associated with those experiences. This embodied lived context is perhaps especially relevant when considering experiences that are closely entwined with concerns of body image and physical perceptions of being-in-the-world (Merleau-Ponty, 1962). In being with the participant one is able to get as close as possible to these personal experiences. This approach however acknowledges that this is a necessarily interpretative endeavour. It is therefore pertinent that reflexivity was practiced throughout the whole research process. This involved challenging taken-for granted perceptions and reflecting on my own experiences in a research logbook at all stages of the research.

### Participants

Eight participants were purposively sampled through social media. Members of online UK based IE support groups on Facebook were targeted. Participants either responded to an advertisement on a support group feed or were contacted through a private message.

The eight participants formed a relatively homogenous group. All participants were female, European and Caucasian and were aged between 26 and 48 (mean = 33 years). All participants had been practising IE for at least 1.5 years. Practice was defined as an active engagement with the IE principles. All participants confirmed that they did not have an active eating disorder. The sample size is normative for an IPA study where research focuses on the detailed analysis of each case. In the reporting of results, pseudonyms were used (Table 1).

**Table 1.** The participants.

Anonymised name (to protect identity)	Age	Time spent practising intuitive eating (years)
Fay	33	4
Jessica	28	2.5
Margaret	31	3
Deborah	34	3
Jo	48	1.5
Sarah	27	3
Daisy	40	1.5
Beth	26	3

### Procedure for data collection

Data was collected through semi-structured interviews. The interviews took place online via Skype and lasted approximately 1 hour. Interviews were recorded with the consent of the participant. All data was stored in accordance with Data Protection Act (2018) and GDPR (2018). The interviews followed an interview schedule devised through exploratory preliminary readings (see Appendix 1). Questions were designed to explore participants lived experiences of the process of IE. Questions were based around the following three areas: general experiences of IE, challenges experienced while learning to eat more intuitively and how these challenges were/are negotiated. The schedule provided structure but allowed a flexible approach that followed the concerns of the participant. Each interview began with a brief introduction to the research and its aims. This was followed by introductory questions which progressed to more in-depth questions designed to explore the participant’s personal experience of becoming an intuitive eater. The participants were encouraged to reflect in detail about their experiences and the challenges that they faced.

Ethical approval for the study was secured through the relevant university. Prior to participation, participants were provided with participant information sheets which outlined the purpose of the study and what participation would involve. Consent forms were also administered which clearly outlined the participant’s right to withdraw themselves and their data from the research at any time before the data was analysed. The consent form also guaranteed the participants anonymity and confidentiality except in the case of protecting individuals from harm. Furthermore, following participation, participants were provided with a debrief sheet which encouraged participants to contact the researcher and/or supervisor with any concerns and provided contact details for relevant helplines should they experience any distress as a result of participation. An awareness of the power discrepancy between researcher and participant was maintained at all times.

### Procedure for analysis

Interview recordings were transcribed verbatim. All participants were provided with the opportunity to review their interview transcript. Data was analysed using interpretative phenomenological analysis (IPA). Analysis closely followed Smith et al.’s (2009) six stage process. Analysis began with close readings and re-readings of the first case and initial noting. This involved line-by-line analysis of the participants experiential concerns and understandings of their experience. By reflecting on my own initial assumptions and judgements I endeavoured to bracket off my own preconceptions in order to actively engage with the data with an open mind. Drawing from these initial exploratory notes, emergent themes were identified. These themes were organised into groups of connected themes with the aid of visual and creative devices. This process was repeated for each case.

Following the independent analysis of all transcripts, patterns were established across cases, developing cross-case superordinate themes that explored convergence and divergence between participants. This process of sustained reflection involved a dynamic circular process that moved between part and whole while maintaining an ideographic focus from within the participant’s experience (Smith et al., 2009). These superordinate themes, along with relevant extracts from interview transcripts were documented in a master table. This master table was then translated into a narrative account supported by extracts from each participant.

## Results

Four related themes were identified. Firstly, participants overwhelmingly described IE as an ongoing counter-cultural process. Given the subversive nature of IE and pervasiveness of diet culture, IE was described as an ongoing process that required a life-long practice of rejecting diet culture. Secondly, participants described the lack of understanding and judgement that they encountered during the process of becoming an intuitive eater. For a number of participants this resulted in an experience of isolation. Thirdly, participants described how they resisted the dominant context of diet culture. Strategies included participating in an alternative supportive community online, engaging in activism and protecting themselves from others. Lastly, all participants described greater freedom to make self-determined choices. This was closely related to an increase in headspace since starting the IE process. This freedom had broader impacts that extended beyond participants’ relationship with food, their body and exercise. A number of participants described the transformative influence of this freedom that enabled them to more fully embrace themselves in a direction of self-actualisation. See Table 2 for a summary of superordinate and subordinate themes.

**Table 2.** Summary of superordinate and subordinate themes.

Superordinate themes	Subordinate themes
An ongoing process of rejection	Counter-cultural nature of IE
	Pervasiveness of diet culture
	Traps and seductiveness of diet culture
	Repetitive rejection/unlearning of diet culture
	Process ‘forever’
Perceived judgment of others	Lack of understanding
	Fear of judgement
	Experience of weight stigma/bias
	Experience of isolation
Strategies of resistance: community, self-protection and activism	Participating in online communities
	Engaging in activism
	Self-protection from other people
A process of self-actualisation: ‘unleashing our whole selves’	Mental struggle while dieting
	Increased headspace as a result of IE
	Listening to body and trusting body’s needs
	Self-determination
	Developing confidence and freedom to be self
	Development of other interests and hobbies

### An ongoing process of rejection

Participants described IE as a counter cultural process that required a process of unlearning, undoing and rejecting diet culture. Participants emphasised the pervasiveness of diet culture and highlighted the seductive nature of the related thoughts. As such IE was described as a perpetual process without a finite completion point.

All participants positioned IE in opposition to diet culture. IE was described as ‘counter-cultural’, (Sarah), ‘genuinely anti-mainstream’ (Margaret) and contrary to inherited social norms. The counter-cultural nature of IE was highlighted by descriptions of the pervasiveness of diet culture. During the process of becoming intuitive eaters, the participants became more aware of diet culture and were shocked by its pervasiveness. As Margaret said:

Reject[ing] the diet mentality, I mean that was hard and that was definitely what made me open my eyes to everything because I was like ‘what’s diet mentality?’ and then you’re introduced to like diet culture and you’re like God it’s everywhere!. . . Diet culture comes from every angle everywhere, from pop ups on your laptop to everything, media, so it’s really hard [. . .] Everything around you is anti-intuitive eating.

Margaret’s assertion that diet culture is ‘everywhere’ and ‘everything’ emphasises the ubiquitous nature of diet culture. This was further emphasised by Jo who said:

the wider context is so powerful, pervasive and long-established, it’s just the air we breathe and [. . .] recognising the pervasiveness of it has shocked me to my core.

This extract emphasises the power of this biopedagogy that infiltrates all areas of life. The description of diet culture as the ‘air we breathe’ is a powerful metaphor that accentuates the inescapable nature of diet culture that Margaret described as ‘coming from every angle everywhere’. It also implies an unconscious nature of diet culture that explains both Jo and Margaret’s shock when discovering its pervasiveness. In addition, the extracts emphasise diet culture as a present challenge. Margaret’s use of both the past tense ‘that was hard’ and the present tense ‘it’s really hard’, suggests that while Margaret has attempted the difficult process of rejecting diet mentality, this process of rejection is very much ongoing.

The inescapable pervasiveness of diet culture was also echoed by Jessica and Deborah who both said that they feel ‘surrounded’ by diet culture. This sense of being surrounded by diet culture infiltrates all parts of participants’ lives including their relationships with family, friends and work colleagues. For example, Deborah stated that her friends and family are ‘constantly talking about like weight or like putting on weight or needing to lose it’, while Beth and Jessica spoke about the pervasiveness of diet culture at work. Given diet culture’s omnipresence, it is unsurprising that several participants explained that thoughts of diet culture often return. Deborah described ‘long moments where I do slip back’ while Beth described the mindful process of watching diet thoughts but not acting on them. Several participants expressed the seductiveness of diet culture and a fear of being ‘sucked back in’ (Jo). As Fay stated:

I think um there’s so many traps, you get so easily get sucked into diet culture again. . . there is still every now again times when I really think about size 10, which I probably never will be, and I’m accepting of most days but every now and then I’m just yeah, feeling a bit down, um thinking that there might be an easy way to get to this goal weight um yeah, that’s seductive.

Fay emphasises the ‘seductive’ allure of diet culture and highlights the challenge of ‘accepting’ her body given the ‘many traps’ of diet culture. This description implies that the threat from diet culture is ever present but is especially dangerous when Fay feels ‘down’. As a result of this continual threat from diet culture, participants described IE as a continual process of unlearning. As Margaret said:

for me at the beginning, rejecting diet mentality, I was triggered all the time, I was just like omg it’s everywhere and eurgh and now it’s a lot easier, but yeah it is a long long journey. . . for me it took and it takes a long undoing and unlearning of everything, yeah, so it is a life-long thing definitely.

Similarly, Deborah said:

I think that it will probably be a process forever, I mean it would be wonderful if that would happen but I think there is so much history and undoing in my psyche I guess that umm that I’ve been just brought up in a world which has that sort of thin ideal, thin health ideal and, and you’re just surrounded by it all the time, and I think as much as you sort of try and stay away from it, it comes back, so it is a constant like undoing I think. Umm so yeah, I think then it is an ongoing process.

Margaret and Deborah describe the continuous mental struggle against diet culture. For Margaret, this struggle has lessened, but is very much still ongoing. Margaret and Deborah emphasise the need for ‘undoing’ and ‘unlearning’ of diet culture in the face of an all-pervasive ‘world’ of diet culture, highlighting the enormity of this process. They both envisage IE as a perpetual process of unlearning. The use of the word ‘undoing’ by both Margaret and Deborah implies the demanding and perhaps destructive nature of this process which requires an unravelling of years of ingrained thought processes.

For Jo, this process of unlearning involves inoculating yourself against the context of diet culture. Jo said:

So yeah there’s you, you you’ve got to inoculate yourself against [diet culture], you’ve got to accept that the context is not supportive of what you’re doing and the general public opinion is not supportive of this, um and do it anyway and that takes a lot of strength.

The need to inoculate herself suggests that Jo feels a strong need to protect herself from the cultural context of diet culture. While inoculation might give the impression of a conclusive immunity from the seductiveness of diet culture, Jo’s acknowledgement of the unsupportive context and strength needed suggests that this requires a persistent concerted effort. Indeed, Margaret emphasised that rejecting diet mentality does not result in immunity. In describing the IE process, Margaret said:

I guess, I feel like it’s [IE] saying reject diet mentality but it’s not saying be immune from it so I think, I think they’re two different things, I think you can reject diet mentality and you can reject fatphobia but it doesn’t mean it doesn’t hurt you, it doesn’t mean that you’re immune from all of those messages [. . .]

I don’t think that you can just reject it when it comes, as a woman, from every angle everywhere, from pop ups on your laptop to just everything, media, so it’s really hard.

This suggests that while rejection is possible, for Margaret this rejection is partial and perpetual and does not entirely protect her from the distressing effects of diet culture. Therefore, the process of IE is an ongoing process of rejection. For Daisy and Beth, this was associated with concerns that they might never fully reach an envisioned destination related to IE. Daisy said:

it is 100 per cent for me an ongoing process and I, I don’t, actually I was thinking about this just before you called, I don’t know that I’ll ever be fine if that makes sense, I just, I think, I think it’s such an ingrained habit in me.

This extract suggests that for Daisy rather than there being a distinct point of completion, IE is experienced as an ongoing process that is never fully resolved. While this might sound negative, for Fay this understanding of the processual nature of IE is comforting. She said ‘I think erm reminding myself that it is a process that is probably never over. I think that helped a lot’. For Fay this acknowledgement of the continual process of IE has helped her, perhaps in the sense that this recognises the unsupportive cultural context in which the process of IE takes place and the strength required to grapple with this context.

### Perceived judgement of others

Participants spoke in depth about the lack of understanding and judgement that they experienced from others. This resulted in feelings of isolation, which many participants described as the most challenging part of IE.

Fear of judgement and lack of understanding from other people was especially acute at the beginning of the IE process when participants described their newly developed beliefs as unstable and ‘fragile’. Sarah said:

I didn’t tell anyone else basically [about IE], umm because I didn’t, I was just really scared people would be like ‘no, you’re wrong.’ Like it was so new to me and my belief and it was so fragile that I couldn’t really like put it out there to anyone else

This fear was often grounded in experience and the majority of participants described difficult conversations with family and friends as well as medical professionals and psychological therapists. For example, Sarah recounted problematic and stigmatising conversations with doctors who praised her disordered eating behaviours, while Margaret and Fay described the difficultly in accessing a therapist who was open to the IE approach and did not engage in weight bias. For Jo, this lack of understanding manifested in a fear that her friends might think she was ‘mad’, ‘batty’ of had ‘lost my marbles’. This highlights the subversive nature of IE, which might be perceived by other people as ‘mad’. Deborah and Beth similarly explained how they didn’t have any friends that ‘really really get it’. In this way participants effectively censored themselves, choosing not to discuss IE with others. These accounts also imply a certain perceived unwillingness on the part of others to listen and understand. For several participants this lack of understanding and communication translated into a feeling of isolation. As Sarah said:

so the fact that it’s counter-cultural I think it, is really difficult to talk to people about it. . . because it’s counter cultural it’s very difficult to explain [. . .] I think that that’s pretty difficult because I think it can feel quite isolating and I think then when you have difficult moments you feel, yeah, isolated.

Sarah explains how IE’s opposition to mainstream culture leads to difficulties in communication which can lead to a feeling of isolation. For Margaret, rejecting diet culture and embracing IE in a community where ‘everybody around you is talking about calories and diet culture’ resulted in a feeling of being on the periphery:

I just felt like intuitive eating at some point kind of kept me on the outskirts, because everyone would talk calories and all that stuff and like I don’t want to join in with that kind of conversation but then if you’d be like ‘have you heard about intuitive eating?’ and it would go quiet, you know I kind of felt I didn’t really have the community and I know it sounds really odd but when everybody around you is talking about calories and diet culture, as human beings I think we want to fit in so I sometimes get drawn into that, but mostly I’m not like that. Does that make sense?

Margaret describes a distinct feeling of ostracisation and feels consigned to the ‘outskirts’ of society. Margaret alludes to the stigmatising nature of opposing diet culture, a stance that is met with silence. She grapples with the desire to fit in and suggests a certain strength or resolve is needed to resist the human need to conform. Her sense of ostracisation is further emphasised in the next extract:

the reasons why I felt quite ostracised is because I didn’t like feeling that, I guess everywhere is so fatphobic, it felt like every. . . it felt. . . in my mind it created this idea that outside is dangerous and hostile and hated people who were in bigger bodies and the only safe place was not there.

Margaret’s ostracisation by the culture that she used to be part of results in a fear of a ‘hostile’ and ‘dangerous’ world. Her use of the phrase ‘the only safe place was not there’ suggests that Margaret was unable to name somewhere that she did feel truly safe. This experience of isolation was threatening and distressing for Margaret. For Jessica, Sarah and Beth, this sense of being isolated and ‘doing it on your own’ (Beth), is the most challenging part of IE. As Jessica succinctly stated, the most difficult part of IE is ‘being surrounded by people who are not [engaging in IE]’. This emphasises the challenge of opposing the culturally normative beliefs about dieting and weight.

### Strategies of resistance: Community, activism and self-protection

Participants described how they resisted the dominant cultural context, negotiating the challenges of IE in three ways: discovering or creating online community, participating in activism and protecting themselves from anticipated judgement from other people.

#### Creating community

Given the sense of isolation and perceived judgement that participants described, the importance of creating a sense of community in other spaces, most notably online, was stated. All participants spoke about the important role that social media played during the process of becoming an intuitive eater. Deborah said:

I think having that, having, following on Instagram those that are, of similar mind or getting there or you know at least not promoting diet culture umm yeah I find that all super helpful because there are just like, yeah, elements of things that I love my friends but they just don’t get it.

For Deborah Instagram provided an understanding that she was not able to get from friends. Several participants described the process of creating a safe supportive environment on Instagram. Fay, Deborah, Sarah and Jess explained how they unfollowed accounts that promote, applaud or depict weight loss stories and disordered eating and began following individuals and groups that are IE or body positivity based.

For Margaret, this search for community led her to create her own online community group on Facebook that focused on body acceptance and organised group meet ups aimed to support each other. Margaret explained that ‘that was huge, like, making me feel less alone’ and was ‘one of the biggest things in my kind of recovery’.

#### Engaging in activism

Participants also reported actively challenging other people’s beliefs. A number of participants described the process of challenging fat phobia or discrimination. In particular, this involved questioning and reviewing language use. As Jessica said:

if I’m having a conversation with someone it is definitely, like if something spiked me then I will like come forward and say that, like you don’t mean lazy, you mean overweight and that’s different, but normally it’s just an underlying thing that is just like part of who I am now.

Jessica describes challenging normalised fatphobic beliefs that equate fatness with laziness. This confrontation of fatphobia in others was illustrated by several participants and demonstrates active attempts to resist pervasive stereotypes propagated by diet culture. In addition, Jessica implies that this activism has become part of a new self that has been formed through this process.

For some participants, activism acquired a more corporeal interpretation. Deborah, who works as a yoga teacher, explained how challenging diet culture was not just achieved verbally, but also ‘by being:’

I’m really enjoying challenging that [diet culture] and also not just challenging that through what I’m saying, but also showing them that that’s like, by being, like showing in action [. . .] I guess because I, as I said though I’m not fat, I’m like erm a size where I can you know buy clothes very easily, but I’m, I’m quite different from a lot of stereotypical yoga teachers [. . .] I think with people seeing me who, you know, isn’t maybe the stereotypical uber skinny yoga teacher, they’re able to maybe feel a little bit more like ‘ah I can, she can do that, she’s really strong, I can do that too,’ that kind of thing and I’ve had that a little bit with some of the people I teach to or some of my following um that it’s sort of yeah, I guess that’s what I mean by kind of showing it by being me [. . .] maybe they’ll even see someone that challenges that and they’ll go ‘oh maybe that means I can challenge that too.

As a yoga teacher Deborah feels that through ‘being’, by simply existing unapologetically in her body, she is able to challenge discourses of what a yoga body should be and give other people the confidence to explore what their body can do regardless of its appearance. Deborah explained that this is particularly significant in the yoga sphere where the stereotypical body is thin. Deborah says that she enjoys this performative resistance and there is a sense of empowerment in the tone of the extract. Indeed, Deborah implies that this process of ‘being me’ has enabled her to more fully embrace herself.

This embodied activism was also experienced by Jessica who explains how bodies can be recognised as a statement:

It feels like you are making a statement with your body without saying anything. Like how dare you be happy as a woman if you are wearing a size large item of clothing.

Similarly, Sarah said:

Specifically, if you are a plus sized person, like remembering that liking your body and eating what you want is a really radical act in itself.

This embodied activism exudes a fighting spirit or strength in the face of diet culture and implies that participants glean a collective sense of empowerment from these embodied acts of resistance.

#### Self-protection

Finally, while all participants described instances of activism, they also all described a process of self-protection from the judgement and opinions of other people. Participants described a selective process of deciding who to engage with as well as developing coping mechanisms to cope with certain difficult situations. Daisy explained that she raises the subject of IE only when it is ‘appropriate and necessary’ while Fay and Beth explained that if ‘someone’s just so fixed in their way’ it is better to ignore them and focus on oneself. Beth explained that ‘if there’s talk at work or jokes and things about it, I don’t always raise it, but I just don’t take part in it’. Sarah explained this selective process further:

In the last year probably year and a half I started to talk to close friends about it, and now I do talk about kind of fat phobia and social justice with people but it’s still very much from a kind of self-protection point of view. Like I very much judge who I have the conversation with, who I trust to be respectful, umm and I don’t, I don’t engage on a lot of stuff [. . .] I do have friends who still have very disordered relationships with food, and I don’t really feel able to go there with them.

This extract shows how Sarah carefully determines who it is safe to talk to. Those who she deems respectful are considered safe, while others who remain entangled in diet culture are considered unsafe. For a number of participants, the choice not to engage involved a process of acceptance acknowledging that, as Daisy said, ‘it’s not always my place to, to fight it or educate people’. Hence Daisy highlights the importance of maintaining a balance between speaking out and protecting the self so that ‘it doesn’t feel like it’s constantly a thing’. This balance of acceptance and activism protects participants from the misunderstandings and judgement of others and conserves energy.

Relatedly a number of participants described strategies for self-protection when faced with diet culture and anticipated judgement. When faced with diet culture conversations, Sarah said:

If people start talking about kind of dieting and food and stuff I will kind of opt out, I have said to people before like ‘I have a history of disordered eating, I don’t want to speak about that’ if people are like ‘what’s wrong, what’s going on?’ But I don’t really go into any more detail. Umm I think that tends to scare people as well because as soon as you say eating disorder or disordered eating people are like ‘oh ok, ok’ [laughs].

This extract shows how Sarah is able to opt out and even shut down diet culture conversations. Sarah utilises other people’s anxiety regarding disordered eating to close down conversations in order to protect her own beliefs from judgement. Jessica explained how she copes with anticipated judgement regarding her weight gain:

I definitely put on weight since I started and um it’s definitely a bit of a . . . like a head screw when you meet someone who you haven’t seen for a long time, because I feel immediately like saying to them, ‘oh like, don’t mention my weight, I know that I have put on weight and I’m fine with it [. . .]

Umm I might make a joke of it and be like ‘hey, so I’ve put on loads of weight but I’m really good at hugs, do you want hugs?’

This extract reveals both Jessica’s fear of being judged and her attempt to pre-empt judgement. This pre-emption implies a desire to retain some kind of control over anticipated judgements. It might be that this control provides a sense of protection from unforeseen comments and the pain that these judgements might cause.

### A process of self-actualisation: ‘Unleashing our whole selves’

All participants clearly described a freeing of headspace since starting the IE process. Through learning to listen to their body’s needs and trusting their body, participants described greater freedom to make self-determined choices both in relation to food, exercise and more widely. In addition, this increased headspace provided opportunities for other interests and hobbies to develop. For a number of participants, these shifts had implications for their identity and enabled them to embrace themselves more fully.

Participants’ descriptions of their relationship with food before starting the IE process overwhelmingly emphasised the near constant mental calculations and evaluations of food choices. Jessica quantified these thoughts, estimating that she ‘used to think about food probably for 15 minutes every hour’ while Fay said that 80% of her mental space used to be consumed by thoughts of dieting, her body and food rules. Emphasising the persistent nature of these thoughts, Daisy said:

My brain which was just constantly full of do this, don’t do this, do this, don’t do this, constantly full of rules that weren’t healthy and so those rules that I was placing on myself were just causing me mass amounts of anxiety around ‘well can I eat this, I don’t know? Is this good for me? Is this bad for me? What should I have? So I really want that but I can’t have that, that’s not allowed.

A very similar mental struggle was described by all other participants. As Jo said:

I never stopped thinking about it, never because I was thinking from the minute I got up in the morning I was doing calculations in my head or on a bit of paper about right, ‘well I’ve had that so and this is what I’m having for tea, so I’ve planned what I’m having for tea, so what does that leave me to make sure I’m fuelled during the day.’ Well I wasn’t thinking about fuel, I was thinking about what am I allowed, you know, this amorphous, invisible thing was allowing me to have things. So yeah, I never stopped thinking about it.

The oppressive nature of these persistent calculations and evaluations which allow little time or headspace for the consideration of other thoughts and interests is clearly communicated in these extracts. Indeed, Jo later described this experience as being on a ‘hamster wheel’, emphasising the circularity and restrictive nature of these thought processes. Relatedly, Jo states that these constant thoughts ‘kept me down and kept me small’ to the extent that looking back she describes herself as a ‘diminished person’. In addition, the use of the word ‘allowed’ in both extracts emphasises the external locus of control. For Jo diet culture assumes an ever-present controlling external ‘amorphous’ force that restricts both her food intake and her sense of self. Sarah similarly associated diet culture with a lack of freedom. She said, ‘there was so much diet culture noise in my head about it, so it never felt like a kind of free choice’.

These descriptions are in stark contrast to how participants described their relationship with food after starting the process of IE. Participants described a simplification of their relationship with food. Beth described her relationship with food as ‘hassle free’, while Jessica described it as ‘a lot more chilled’. Jo explained the change to her relationship with food further:

it’s probably the thing I notice more now about the IE process is that you just don’t think about it. I’m hungry, I eat, I really enjoy my food, I love cooking, I mean I still read good food magazine and [food] porn, you know I love looking at it, thinking about it but once I’ve eaten I don’t think about it until it’s nearly tea time, and oh it’s time to cook, you know [. . .]

My body is just telling me now what it wants. And some days what is wants is a pizza and some days what it wants is pickled beetroot. I’m pretty comfy to go with that now.

These extracts describe a more relaxed and carefree attitude towards food devoid of constant questioning and evaluation. There is a lightness to Jo’s description that suggests that the mental anguish experienced before has dissipated. Jo describes a process of listening to her body’s needs and trusting those cues which was an important shift for several participants. The extracts below from Sarah outline this increased trust in her body and her strong belief in the benefits of this trust.

I actually trust my body now, so if I’m craving something, I trust that it’s what my body needs and wants. [. . .]

I just truly believe that the best thing for my mental and physical health is to do what feels good for my body. I actually truly believe that and I just never used to believe that.

These extracts indicate a transition from being controlled by external messages and rules of diet culture to an intuitive internal regulation of food intake and imply the reassertion of self-determination. This sense of self-determination was especially apparent in participant’s relationships with exercise since starting IE. For Sarah, Daisy and Fay this is characterised by the ability to opt out. Daisy said:

Like I realised I just hated a certain type of work out, so it wasn’t even just the eating, it was. . . I hate HIIT workouts, I hate putting my body through burpees and squat jacks, I hate it! So now I just refuse to do them, I’m just like no, life’s too short, I can exercise in other ways, I don’t really care as long as I’m moving and active and doing something. So, it was just kind of, I think just allowing myself to say no, just I don’t want to, [laughs] that’s ok.

This extract highlights Daisy’s newfound ability to listen to herself and to say ‘no’ to forms of exercise that she does not enjoy. She emphasises that ‘that’s ok’, reiterating that she has given herself permission to dislike HIIT and act accordingly. This permission implies a sense of acceptance and trust in herself. This ability to listen to oneself and say ‘no’ is also expressed by Sarah:

Your body knows what it needs and like, for example I did a barre class yesterday online because people have been raving about it and I just hated it so I just stopped doing it. Like it just felt punishing, it didn’t feel like what I wanted to do and in the past I would definit. . . and there were definitely some thoughts of like ‘oh, you’re too unfit, that’s why you’re not enjoying it, if you were fitter you’d enjoy it, like you’re obviously not strong enough’ or ‘it’s because you haven’t been doing x, you haven’t been doing y,’ and I was like ‘no, I’m not going to do, I’m not enjoying it.’ And the ability to just opt out of stuff and be like this isn’t like working for me, it’s not, it’s not fulfilling what I need it to fulfil, it’s just very liberating, I think.

Despite the presence of critical diet culture thoughts, Sarah exercises her ability to ‘opt out’. Sarah listens to what the barre class felt like (‘punishing’) and decides that it is not what she needs. Despite the influences of diet culture, Sarah maintains the ability to listen to herself. As implied by Daisy, Sarah describes this practice of attending to one’s likes and dislikes as ‘liberating’. This liberation denotes a freedom from the exterior influences of diet culture. For Deborah, this ability to listen to one’s body and make self-determined choices has enabled a shift in her rationale for exercising:

I exercise for different reasons now. Err I probably used to exercise to burn calories and lose weight um and that was probably also part of it. Whereas now I can choose things I like to do or I can set goals for myself that I actually want to do, not feel like I have to do. And it’s normally coming from a place of I want to be really strong or I want to be able to lift this certain weight or do this particular yoga position or whatever, so, yeah I think it’s given me the space in lots of different ways.

Deborah’s emphasis on doing things that she ‘want[s] to do’ rather than those she feels she ‘has to do’ again implies a shift from external control to internal control. Like Daisy and Jo, Deborah is better able to decide how she wants to exercise free of external compulsion or interference. This ability to listen to oneself implies a sense of empowerment and confidence in determining one’s own actions. As Deborah implies, this sense of empowerment is not simply limited to exercise but has provided her with ‘space in lots of different ways’. Sarah also explains how the process of IE has had implications that were broader than her relationship with food. She describes a process of developing confidence in herself and her own choices:

Ummm so it’s been a lot of it has not really been about the food, it’s been about addressing those underlying things about not letting other people define my self worth, having the confidence to make my own decisions and, and the fact that some people are not going to like that. And being able to deal with that [. . .]

Umm it, I think it was the first, it was genuinely like the first experience that I’d had of making a decision that went against like a societal norm as well. Like just being like actually no like you’re, you’re like I think you’re wrong and I’m gonna do what I think. It was my first experience of doing that ever because I’ve always been such a people pleaser and a perfectionist, so, and then once I’d done that in one area it kind of opened up others as well.

Sarah echoes Deborah’s assertion that IE has opened up space in a broader sense. For Sarah, this has included reclaiming the evaluation of her self-worth, establishing confidence in her own decisions and developing courage to go against social norms and assert her own opinions. The resistance of one cultural norm engendered the ability to challenge other norms. Daisy relates this ability to make one’s own choices with freedom.

There’s sort of more confidence I guess, umm, in, in me being allowed to choose, I don’t know, I was going to use the word freedom because it is just kind of back to that confidence to be free and be, to be you and [laughs] I’m gonna use the phrase again, to do you.

This extract highlights the importance of IE in enabling Daisy to gain the confidence and the freedom to be herself, both ‘to be you’ and ‘to do you’. Both Daisy and Sarah highlight the significance of the IE process in the growth of their sense of self and their ability to be themselves.

Furthermore, a number of participants explained how the increased headspace that they now experience allowed them the freedom to explore other interests and hobbies. Jessica said:

There is definitely space for other stuff because I just don’t think about it anymore. . .it means you can care about other stuff and find other hobbies and stuff, which is nice.

For Jo especially, the freeing of headspace provided her with the time and mental energy to develop new hobbies. Jo said

I’ve taken up hobbies, creative hobbies, I wouldn’t of described myself as a creative person [before], I have taken up sewing, so I make things, umm, I have taken up calligraphy, I’ve taken up painting, I read more, I just, it’s just [laughs] my brain has all this space in it.

For Jo, this discovery of new hobbies due to increased headspace has opened up a new creative dimension of herself. For other participants, including Margaret, Sarah, Beth, Deborah and Jessica, the freeing of headspace has enabled them to engage in social justice issues. As Sarah said:

I think about dieting and umm eating disorders and they make you really really self-focused, like cause that is all you think about and everything else is kind of on hold and I think that particularly, the yeah, the last year when I’ve felt so much more settled in my food and just psychologically, I’m starting to actually like look up and out more and think about social justice issues, like educating myself on stuff like, like body justice and systemic racisms and all this stuff that I never really had the headspace for before.

For Sarah, IE has transformed her outlook from inward looking to outward looking. This reveals an interesting dynamic. By turning inward and listening to the body one paradoxically has the space and freedom to adopt a more collective focus that ‘look[s] up and out more’. In this way, through new interests and hobbies, participants were able to expand their knowledge and skills in a self-actualising direction. This freedom to develop new interests along with an increased sense of self-determination had impacts for participants’ sense of self and acceptance of that self. Indeed, a number of participants stated the personally transformative role of IE in their life from affecting their world view and values to creating, as Deborah said, ‘a new way of being’. This extract from Jo encapsulates this process of acceptance and self-actualisation:

But the more I think about it, the more I do think that this is, this is part of reclaiming. . . And unleashing our whole selves. That, that’s the thing, it’s the minimisation of the head space and imagination by keeping you completely constrained by a set of rules that are just mad. I keep saying mad [laughs]. Um but that, what is it, that quote, is it from Naomi Woolf in her. . . book. . . in the beauty myth, about hmm errm keeping the female population hungry and quietly mad, I get it! [laughs] I really do and I didn’t when I first read it but in seeing how I’ve changed and what I have at my disposal, and I didn’t think I was a diminished person before, I thought I had a lot of personal resources at my disposal but I have a lot more now.

This extract brings together the sense of constriction cause by a minimised headspace and the freedom that IE has brought to the participants. It demonstrates how Jo’s awareness of her sense of self has grown and developed from a ‘diminished person’ to someone with greater personal resources. This extract also accentuates the sense of strength and resistance that is demanded by the IE process in order to fully unleash one’s whole self from the constraints of diet culture. Through continually resisting and rejecting diet culture, participants were able to more fully embrace themselves.

## Discussion

This research contributes to the scarce qualitative research exploring women’s experiences of learning to eat intuitively. The participants in this study illustrate the challenges of IE, emphasising the dominant cultural context of diet culture and the resistance required to challenge this context. Through the IE process, participants described an increase in headspace, freedom and self-determination. Four interconnected themes were identified: IE as an ongoing process, perceived judgment of others, strategies of resistance and processes of self-actualisation.

While quantitative research has largely neglected the influence of culture in the process of IE, this qualitative exploration provides a distinctly contextualised account of learning to eat intuitively. Participants overwhelmingly described the cultural challenges faced during the process of IE, most notably, the pervasiveness of diet culture and judgement from other people. The challenge of negotiating and resisting diet culture was evident across all four themes and is concordant with broader research that explores the dominant sociocultural discourses that prescribe information and directives regarding, food, health and bodies (LaMarre and Rice, 2016). The findings correspond to Barraclough et al.’s (2019) research who identified significant social and environmental barriers to the IE process. The participants in this research similarly encountered a significant lack of understanding from friends, family and work colleagues. The significance of the perceptions of others is also expressed in the acceptance model of IE where the perceived lack of acceptance of others impacts directly on one’s acceptance of self and has implications for eating patterns (Avalos and Tylka, 2006).

This research, however, alludes to a broader and more potent system of social and environmental challenges. The ubiquity and power of these external challenges was repeatedly emphasised by participants and IE was positioned in direct contrast to the dominant culture. Indeed, participants emphasised the counter-cultural nature of IE as the most salient difficulty experienced while learning to eat intuitively. This sense of challenge is unsurprising given the monetary power and vast customer base of the diet industry. The concept of biopedagogies is useful in this regard because it highlights the power of these dominant socio-cultural discourses (Wright and Harwood, 2012). Participants described the strength that was required to continually reject, unlearn and undo these discourses. This broader interpretation of sociocultural barriers to IE has been similarly highlighted in eating disorder recovery research. Participants in LaMarre and Rice’s (2016: 143) research described the process of recovery to ‘normal’ eating as ‘counter-cultural’. Thus, while Barraclough et al.’s (2019: 3) research found that participants positioned IE as an ‘alternative’ to dieting, in this research participants positioned IE in direct opposition to dieting and culture more broadly.

While the power and pervasiveness of the dominant biopedagogies of diet culture was emphasised, participants described several strategies of resistance. Indeed, the notion of biopedagogies derived from Foucault’s (1978) theory of biopower necessitates the recognition of the scope for resistance. The importance of resistance is also highlighted by the IE acceptance model where resistance to adopt an observer’s perspective of one’s body is associated with body acceptance and IE behaviours (Avalos and Tylka, 2006). This research extends this conception of resistance and emphasises that rather than being passive subjects shaped by discourses, participants negotiated and resisted biopedagogies of diet culture. This research outlines three main strategies: the creation of or participation in a supportive online community, engaging in activism and strategies of self-protection. In some instances, these resistances incorporated a corporeal expression. This is perhaps unsurprising given the corporeal nature of biopedagogies. Participants described an embodied resistance through which they attempted to alter discourses associated with certain bodies. This performative resignification of the body was closely related to participants sense of self and appeared to play a role in empowering participants to more fully embrace themselves.

This sense of empowerment was reiterated by participants in their descriptions of a transition from being controlled by external rules of diet culture to an internal sense of control. Participants described how they experienced an increase in headspace, learned to listen to themselves and developed trust in their body. This supports Schoenfeld and Webb’s (2013) research which suggested that the internal focus of IE is related to an alignment with one’s internal values in the domain of food consumption and emphasises the oppressive self-disciplining nature of dieting. Significantly, however, this current research implies that this sense of embracing one’s values extends beyond relationships with food, bodies and exercise. Through an increase in headspace and internal control, participants described how they have more confidence, feel freer and have developed new interests and hobbies. This freedom from the self-disciplining imperative of diets enabled participants to more fully reclaim, unleash and embrace their full selves. These descriptions are reflective of Barraclough et al.’s (2019) research in which participants described a realignment of values, a re-examination of deep-seated views about self and a recovering of lost aspects of self. This supports Avalos and Tylka’s (2006) proposal that IE is an expression of the actualising tendency. In the rejection of external directives related to the biopedagogy of diet culture participants were able to turn inwards and pursue their personal ambitions and potential.

While this process of self-actualisation might imply a principally internalised perspective, participants illustrated more nuanced attitudes. Although participants clearly described the development of an inward connection, they also demonstrated a broader outward inclination and awareness. Participants described a need for community, an increased interest in social justice issues and an engagement in activism. This suggests that while IE encourages the prioritisation of internal experience, this process also engenders a more collective focus that extends beyond the self towards an awareness of other people’s experiences and struggles. This is perhaps reflective of a shift away from diet culture’s focus on neoliberal individualised responsibility towards a more collective view of society.

### Limitations and suggestions for further research and interventions

It is important to be fairly cautious in the claims made from this small sample and it should not be assumed that equivalent findings would be established from other individuals learning to eat intuitively. Despite this, the way in which participants spoke similarly and with such intensity emphasises the importance of the themes outlined. There is a pressing need for more qualitative studies exploring the process of becoming an intuitive eater. It would be useful for subsequent research to explore the process of IE across other purposefully selected samples, for example based on age group, gender, nationality, ethnicity and socio-economic status. Given the significance of the cultural context in learning to eat intuitively, research could also explore how IE is experienced in other cultures where perspectives on food, bodies and health differ. Future research should in particular explore males’ experiences of IE, who are notably underrepresented in IE research. This research could then be compared to the findings of this study. In addition, given the apparent ongoing process of IE, it would also be useful to conduct a longitudinal study exploring the experiences of individuals across different points in the process of IE. This research could explore the changes and constancy of experience throughout the process of IE.

Additionally, research could further explore the proposal that IE is an expression of the actualising tendency, perhaps among different samples. Given the transformative potential of IE described by participants of this study, research should further investigate the potential for IE to positively support self-actualisation. Furthermore, this research was conducted with women with relatively homogenous weights/body size. Only one woman identified as ‘plus sized’ with the remainder describing themselves as ‘straight sized’. While this research endeavours to be weight inclusive, it is also important to acknowledge that individual’s embodied experiences of the world are undeniably impacted by their physical appearance. Thus, further research should explore experiences of IE with individuals with diverse body sizes.

This research also suggests implications for interventions. The findings of this study support the development of a more contextualised approach to IE that acknowledges the often-unsupportive nature of the dominant cultural context and the associated challenges of this. Such an intervention could help those supporting individuals through the IE process to recognise that IE is not a finite process, but an ongoing negotiation with diet culture. The return of diet thoughts during the IE process is not the result of personal weakness but an integral part of the process. In addition, future IE interventions may benefit from encouraging the family and friends of individuals learning to eat more intuitively to engage with the principles of IE. Individuals might benefit from being taught skills on how to discuss and explain the process with family and friends. Given the isolation experienced by participants, IE peer support groups might be helpful in providing further support to individuals. Furthermore, it is perhaps premature, but there may be scope for applied psychologists to embrace the potential for IE to engender a self-actualisation process in clients.

## Reflexivity

From the planning stages of this research I have been aware of the necessity of reflecting on my relationship to this topic. Having transitioned to IE myself this topic is of personal importance to me. During the research process I maintained an inductive focus on ideographic meaning and context. At each stage I reflected on my fore-conceptions and embraced an ‘orientation of openness’ (Gadamer, 1990/1960: 367). Despite this iterative process of reflection, interpretation is inevitably shaped by preconceptions. The resultant research is therefore situated and co-constituted between researcher and participants.

This also led me to reflect on my potential status as an insider as someone who considers herself an intuitive eater. Given the alternative stance of IE, I felt that it would be helpful and equitable to communicate my insider status to the participants. Potential benefits of this status are particularly notable at the initial stages of research where personal experience can be useful in accessing participants and developing meaningful and nuanced interview questions (Hayfield and Huxley, 2015). At times during the interviews, I felt a sense of kinship with participants. This perhaps enabled participants to feel more at ease by reducing the fear of weight-biased judgement. However, this position highlighted the need to maintain an inductive focus on participants’ experiences and to reflect on the ways in which I am similar and different to participants. This reflection was important so as not to assume meaning, mistakenly project my own experiences or inadequately explore participants’ unique experiences. It is also important to acknowledge that the dualism between insider and outsider is artificial since these categories are not mutually exclusive and researchers are never ‘outside’ society (Hayfield and Huxley, 2015). Rather I, as the researcher inhabit a space between, balancing the perspectives of involvement and detachment.

While reflexivity is often considered a disembodied practice, Burns (2003) emphasises the material and textual tensions that are elicited by the body. Burns (2003: 230) retheorises interviews as ‘embodied interactions’ that require embodied reflexivity. Whilst the implications of the body are somewhat limited since this research was carried out over Skype, given that participants spoke in depth about body image issues, internalised fat phobia, societal fat phobia and thin privilege, I feel that reflecting on my embodiment provides opportunities for further analysis. As a straight sized^1^ woman, I too benefit from what one participant called ‘the continuum of thin privilege’. During the interviews with eight different participants the perceived point at which our respective bodies fell on this continuum potentially shaped the subjectivity and experience of participant and interviewer. I am aware that given my relative privilege, those participants in bigger bodies may have questioned my ability to understand their lived experience and perhaps even might have been wary of judgement.

In addition, I acknowledge that this research is shaped by the sociocultural environment in which it was conducted. Throughout the research process I attempted to embrace a self-aware stance, staying particularly attentive to influences of diet culture and weight bias.

## Conclusion

This research highlights the contextual nature of learning to eat intuitively. The counter-cultural nature of IE was identified as the most challenging barrier to learning to eat more intuitively. Given the pervasiveness of diet culture, rejecting this biopedagogy was described as an ongoing life-long process. Participants perceived significant judgement and lack of understanding from others. Participants described several strategies to resist diet culture and cope with the judgement of others. These included participating in online communities, engaging in activism to counter weight bias and methods of self-protection. Furthermore, participants described an increase in headspace, an improved ability to listen to themselves and act accordingly, and the development of new interests and hobbies. This had implications for participants’ sense of self and enabled them to embrace themselves more fully. The findings imply that IE has a wider impact that extends beyond participants’ relationships with food, bodies and health and supports claims that IE is an expression of the actualising tendency.

More research is required to further understand the experiences of learning to eat intuitively in order to support those making this transition. Given the ethical concerns relating to the failure of diets and the related adverse health effects, this research has the potential to benefit many. It hoped that this weight inclusive account of IE will contribute to the paradigm shift required in psychology and more broadly.

## Appendix

**Appendix 1.** The interview schedule.

1. What led you to intuitive eating and why did you decide to begin this process? - How did you hear about intuitive eating? - When did you start the process of becoming an intuitive eater? 2. Can you describe your experience of becoming an intuitive eater? 3. What have you found most challenging about becoming an intuitive eater? - Did you find a particular stage challenging? - Are there any parts of diet culture that have been more challenging to let go of? 4. How have you dealt with these challenges? - Have you employed any coping strategies? - Have you utilised any support groups/networks during this process? 5. How have the people around you responded to your process of IE? 6. Has intuitive eating impacted your life / your general wellbeing? - In what ways? 7. What advice would you provide someone embarking on this ‘journey’?

## Notes

1. The category ‘straight size’ tends to refer to women who wear clothes that are smaller than a UK16.
